# Establishment of a genome editing system and expression of heterologous proteins in the nonconventional yeast *Pichia kudriavzevii*

**DOI:** 10.1002/mlf2.70104

**Published:** 2026-09-29

**Authors:** Jiayin Ma, Yifan Cui, Binru Yin, Cheng Jin

**Affiliations:** ^1^ State Key Laboratory of Microbial Diversity and Innovative Utilization, Institute of Microbiology Chinese Academy of Sciences Beijing China; ^2^ University of Chinese Academy of Sciences Beijing China; ^3^ Beijing CAStar Union Sci‐Tech Ltd. Beijing China

## Abstract

Nonconventional yeast *Pichia kudriavzevii* possesses attractive phenotypic traits for industrial biomanufacturing; however, it remains genetically intractable compared with established yeast chassis. Here, we present an integrated synthetic biology framework that renders *P. kudriavzevii* a programmable production host. We established a highly efficient CRISPR/Cas9 genome editing system in this diploid yeast, enabling marker‐free editing with efficiencies of up to 88% and routine generation of homozygous mutants. By integrating efficient marker‐free CRISPR/Cas9 genome editing and locus‐aware expression strategies, we successfully expressed secretory mCherry and human IgG1 Fc fragment at different loci. Our results demonstrate that *P. kudriavzevii* is a promising expression system for protein production.

Over the past two decades, synthetic biology has transformed a small number of model organisms, most notably *Saccharomyces cerevisiae* and *Komagataella phaffii* (also known as *Pichia pastoris*), into genetically programmable platforms through the development of efficient genome editing technologies and finely tunable gene expression systems^1^. In parallel, a growing interest has emerged in nonconventional yeasts, which often show superior stress tolerance, broader substrate utilization, and higher robustness under industrially relevant conditions. However, the practical exploitation of these organisms has lagged behind their phenotypic promise, largely due to a paucity of molecular toolkits that enable precise and dynamic genetic control.

*Pichia kudriavzevii* displays remarkable tolerance to high temperatures, low pH, and diverse environmental stressors^2^, ^3^, ^4^. We recently isolated a thermotolerant *P. kudriavzevii* strain capable of sustained growth and ethanol production at 37–42°C^5^, highlighting its potential as a next‐generation industrial chassis. However, the broader adoption of *P. kudriavzevii* has been constrained by limited genetic programmability. Although several CRISPR/Cas9‐based genome editing systems have been established in *Issatchenkia orientalis* (a synonym of *P. kudriavzevii*)^6^, ^7^, ^8^, ^9^, the editing efficiencies vary substantially across studies, reflecting differences in the host strain background, Cas9 variant design, and the strategies for single‐guide RNA (sgRNA) expression and processing. Moreover, many existing CRISPR platforms remain dependent on strain‐specific selection markers or are optimized primarily for single‐gene knockouts, complicating marker recycling and multi‐round genome modification in a diploid context. As a result, although proof‐of‐concept genome editing has been achieved, *P. kudriavzevii* still lacks a robust and broadly applicable genome engineering toolkit suitable for systematic metabolic rewiring.

To establish a genome editing system in *P*. *kudriavzevii*, we constructed a CRISPR/Cas9 plasmid based on yeast shuttle vector pPpT4_GAP^10^. As autonomously replicating sequence (ARS) is essential for episomal plasmid maintenance in yeast and can substantially facilitate transformation^11^, we first screened several ARS sequences from different yeast species (Table S1). The primers used in this study are listed in Table S2. When ARS derived from *Kluyveromyces lactis*, *K. marxianus,* or *Komagataella phaffii* was applied, no transformant was observed, while *S. cerevisiae* ARS (ScARS) was found to consistently support episomal plasmid propagation, as evidenced by robust colony formation (Figure S1A). Therefore, the ScARS was used as the backbone for subsequent CRISPR/Cas9 system optimization.

Efficient CRISPR/Cas9‐mediated genome editing requires robust and coordinated expression of both Cas9 and sgRNA. We further evaluated multiple combinations of Cas9 and sgRNA expression cassettes (Table 1). Among all cassettes tested, only construct 8 showed genome editing activity in *P. kudriavzevii*. In this construct, the Cas9 coding sequence was optimized based on the codon‐usage bias of *P*. *kudriavzevii* (Figure S1B), flanked by simian virus 40 (SV40) nuclear localization signals at both the N‐ and C‐termini, and placed under the control of the endogenous *P. kudriavzevii RNR2* (the small subunit of ribonucleotide reductase) promoter^12^ with the *CYC1* (cytochrome *c*, isoform 1) terminator. For sgRNA expression, an RNA polymerase III‐driven strategy was employed using the endogenous *P. kudriavzevii RPR1* (the RNA component of nuclear RNase P) promoter fused with tRNA^Leu^ ^9^, which facilitated efficient sgRNA release from the primary transcript (Figure S1C).

**Table 1:** Combinations of Cas9 and sgRNA expression cassettes tested in *Pichia kudriavzevii*.

Construct	Promoter of Cas9	Cas9	Promoter of sgRNA	Ribozyme‐flanked sgRNA expression
1	P_*HTA*_	HsCas9	P_*HTB*_	HH and HDV
2	P*_HTA_*	PkCas9	P_*HTB*_	HH and HDV
3	P_*TDH3*_	HsCas9	P_*RPR1*_	—
4	P_*TDH3*_	PkCas9	P_*RPR1*_	—
5	P_*TEF1*_	HsCas9	P_*RPR1*_	—
6	P_*TEF1*_	PkCas9	P_*RPR1*_	—
7	P_*RNR2*_	HsCas9	P_*RPR1*_	—
8	P_*RNR2*_	PkCas9	P_*RPR1*_	—

Different promoter–Cas9 configurations were paired with sgRNA expression cassettes driven by the *RPR1* promoter, with or without ribozyme‐flanked designs. Codon‐optimized Cas9 variants derived from *Homo sapiens* (HsCas9) and *P. kudriavzevii* (PkCas9) were evaluated under multiple promoters. HDV, Hepatitis Delta Virus ribozyme; HH, Hammerhead ribozyme.

Genetic transformation systems based upon complementation of orotidine 5′‐phosphate decarboxylase (OMP decarboxylase; EC 4.1.1.23) mutants are widely used for many fungal species, such as *S. cerevisiae*, *Aspergillus nidulans*, *Penicillium chrysogenum*, *A. niger*, *Trichoderma reesei*, and *A. fumigatus* ^13^, ^14^, ^15^, ^16^, ^17^, ^18^. To obtain a genetic transformation system, we deleted the *ura3* (encoding OMP decarboxylase) gene in *P. kudriavzevii* using the CRISPR/Cas9 system. Transformants carrying the CRISPR/Cas9 plasmid and donor DNA were screened on selective medium. PCR analysis revealed that a 2.5‐kb fragment in the wild type and a 1.7‐kb fragment in the *ura3‐*deletion mutant were detected (Figure S2A). Consistent with the diploid nature of *P. kudriavzevii*, heterozygous mutants contained both fragments. Phenotypic assays further confirmed that *ura3* mutants only grew on uracil‐supplemented medium, but failed to grow on medium without uracil (Figure S2B,C). Genotyping of 50 independent transformants revealed that 44 clones carried the targeted deletion, including 15 homozygous knockouts. The generated homozygous mutant of the *ura3* provides a strain for genetic transformation. In addition to the *ura3* locus, we extended our validation to other genomic sites. Specifically, we successfully performed *KU70* gene deletion and confirmed the desired genotypes by diagnostic PCR (Figure S3). In addition, previous results have confirmed that this editing system can modify the glycosylation pathway in *P. kudriavzevii* ^19^. These results further demonstrate that the CRISPR/Cas9 system is effective across multiple independent loci and supports its broader applicability in genome engineering of *P. kudriavzevii*.

To assess the potential application of *P. kudriavzevii* in heterologous protein expression, we attempted to insert the exogenous gene into the genome of the Δ*ura3* mutant by the CRISPR/Cas9 system. In addition to the commonly used 18S rDNA locus, which exists as a high‐copy repetitive array and allows multicopy integration to increase gene dosage and enhance protein expression, the *och1* (outer chain elongation 1) locus was also chosen as the potential integration site. The *och1* encodes a mannosyltransferase responsible for elongation of N‐glycans of glycoproteins to high mannose type of N‐glycan. Previously, we have shown that deletion of the *och1* can block the formation of the yeast high‐mannose type N‐glycan, suggesting a possibility that insertion of heterologous genes can produce glycoproteins with N‐glycans closer to mammalian type^19^.

mCherry fused to the SED1 (stress‐induced cell wall protein 1) signal peptide was constructed under the control of P_*TDH3*_ (constitutive *PkTDH3* promoter). The mCherry expression cassette was independently integrated into either the *och1* or the 18S rDNA locus using the established CRISPR/Cas9 system (Figure 1A). As a result, the secreted mCherry was readily detected in the culture supernatant by SDS‐PAGE (Figure 1B) and confirmed by mass spectrometry (MS) (Figure 1C). Using bovine serum albumin as a standard, the productivity of the expression cassette at the 18S rDNA locus was calculated to be 475.8 ± 0.52 mg/l, while integration at the *och1* locus yielded 297.7 ± 4.65 mg/l of mCherry (Figure 1D). It was noted that the integration at the *och1* locus reduced by ~36% of biomass. Interestingly, when the productivity was calculated as milligram per biomass (OD_600_), the secreted mCherry at the *och1* locus and the 18S rDNA locus was 66 mg/OD_600_ and 68 mg/OD_600_, respectively (Figure 1E). These results suggest that the difference in production at different loci is due to the effect of integration site on growth.

**Figure 1:**
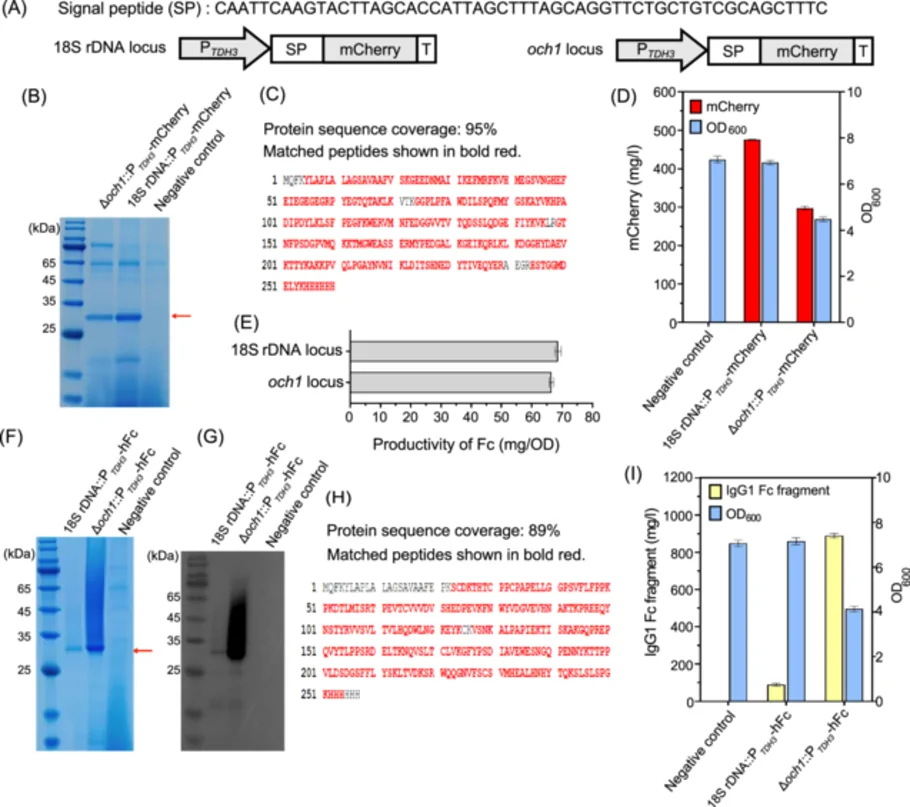
Expression of mCherry and human IgG1 Fc fragment in *Pichia kudriavzevii*. (A) Diagram of the expression box construction and the signal peptide sequence. (B) SDS‐PAGE analysis of the culture supernatant. The mCherry band is indicated by the red arrow. (C) Mass spectrometry analysis confirming the identity of mCherry excised from SDS‐PAGE. (D) Concentration of mCherry at different loci. The values represent mean ± SD of three biological replicates. Statistical significance was determined using one‐way ANOVA (Negative vs. 18S rDNA::P_*TDH3*_‐mCherry, *p* < 0.0001; Negative vs. Δ*och1*::P_*TDH3*_‐mCherry, *p* < 0.0001; and 18S rDNA::P_*TDH3*_‐mCherry vs. Δ*och1*::P_*TDH3*_‐mCherry, *p* < 0.0001). (E) Productivity calculated as milligrams per OD_600_ at different loci. (F) SDS‐PAGE analysis of the culture supernatant. The Fc fragment band is indicated by the red arrow. (G) Western blot validation of Fc fragment secretion. (H) Mass spectrometry analysis confirming the identity of the Fc fragment excised from SDS‐PAGE. (I) Yield of the purified human IgG1 Fc fragment at different loci. The values represent mean ± SD of three biological replicates. Statistical significance was determined using one‐way ANOVA (Negative vs. 18S rDNA::P_*TDH3*_‐Fc, *p* < 0.0001; Negative vs. Δ*och1*::P_*TDH3*_‐Fc, *p* < 0.0001; and 18S rDNA::P_*TDH3*_‐Fc vs. Δ*och1*::P_*TDH3*_‐Fc, *p* < 0.0001). ANOVA, analysis of variance; SDS‐PAGE, sodium dodecyl sulphate‐polyacrylamide gel electrophoresis; T, terminator.

When the Fc expression cassette was introduced into either the *och1* or the 18S rDNA locus, secreted Fc was detected in the culture supernatant by SDS‐PAGE, Western blotting, and MS (Figure 1F–H), and purified with protein A column for quantitative analysis. Although integration of *IgG1 Fc* at the *och1* locus resulted in a 42% reduction in biomass, a higher Fc titer of 889.1 ± 13.11 mg/l (222 mg/OD_600_) was detected, which exceeds the previously reported highest titers for IgG1 Fc (~25–30 mg/l) in yeast^20^. This reduction in biomass may be attributed, at least in part, to disruption of *och1*, which is known to affect cell wall integrity (Table S3). In contrast, integration of *IgG1 Fc* at the 18S rDNA locus resulted in a lower production level of only 88.8 mg/l (12 mg/OD_600_) (Figure 1I). Consistent with these observations, quantative PCR (qPCR) analysis showed that transcript level of the *Fc* gene was lower at the 18S rDNA locus than at the *och1* locus (Figure S4). This locus dependence highlights the protein‐dependent effects of genomic integration sites on secretory expression, and the *och1* locus is a better integration site for IgG1 expression.

A central foundation of this framework is the establishment of a highly efficient, marker‐free CRISPR/Cas9 genome editing system tailored to *P. kudriavzevii*. By optimizing episomal plasmid stability, Cas9 expression, and sgRNA transcription, we achieved high editing efficiencies and routinely obtained homozygous mutants in a single transformation step. Notably, our results also suggest that proper tuning of Cas9 expression is critical for editing performance, as excessive expression may impose a metabolic burden and induce DNA damage stress, thereby compromising cell viability and reducing apparent editing efficiency. In contrast, a more balanced expression level may facilitate efficient Cas9–sgRNA complex formation while maintaining cellular fitness. To enhance heterologous protein expression, we targeted integration to the 18S rDNA locus, which exists as a high‐copy repetitive array. Integration at this locus can result in multi‐copy insertion and potentially increase gene dosage, a strategy widely used in *S. cerevisiae* and other yeast systems to achieve high‐level expression of heterologous genes. Consistent with this approach, mCherry expression was high at the 18S rDNA locus, whereas Fc protein expression at the same locus was lower than that at the *och1* locus, indicating that expression outcomes depend not only on copy number but also on protein‐specific factors such as transcript processing, translation efficiency, and local chromatin context. Notably, the *och1* knockout strain showed modest growth defects, consistent with its role in cell wall mannosylation and maintenance of cellular integrity. These physiological changes could influence overall protein production by affecting biomass accumulation or secretion capacity. Nevertheless, Fc expression remained higher at the *och1* locus than at the 18S rDNA locus, suggesting that locus‐specific effects on transcription, translation, and protein processing may outweigh the moderate impact on growth under the conditions tested. These findings indicate that no single genomic integration site universally optimizes protein secretion in *P. kudriavzevii*, necessitating systematic benchmarking across multiple loci rather than reliance on presumed “safe harbor” sites. Furthermore, *P. kudriavzevii* achieved IgG1 Fc fragment titers of 889.1 mg/l in shake‐flask cultures. Although direct cross‐study comparisons should be interpreted with caution due to differences in strains, constructs, and cultivation conditions, this level substantially exceeds previously reported yeast titers for IgG1 Fc (~25–30 mg/l) and full‐length IgG (~238 mg/l). These results suggest that *P. kudriavzevii*, when combined with an optimized genome editing and expression framework, represents a competitive and scalable alternative to conventional yeast expression platforms.

Importantly, marker‐free homozygous editing enables repeated rounds of genome modification without genetic scarring, a prerequisite for complex pathway construction and chassis optimization. By integrating efficient marker‐free CRISPR/Cas9 genome editing and locus‐aware expression strategies, we provide a blueprint for converting the intrinsic stress tolerance of *P. kudriavzevii* into controllable and scalable genetic function. Our results demonstrate that *P. kudriavzevii* is a complementary and versatile chassis that expands the functional landscape of yeast biotechnology.

Although substantial levels of human IgG Fc fragment were produced, the glycan structures are not yet human type. Thus, future work should expand its application to multi‐gene pathway control and therapeutic protein glycoengineering. In addition, systematic benchmarking against established hosts, evaluation under industrially relevant stress conditions, and exploration of strategies for expressing difficult‐to‐fold proteins (e.g., chaperone co‐expression or secretion pathway engineering) will further strengthen the robustness and applicability of this platform. The genome editing framework described here provides a foundation for reconfiguring the glycosylation pathway of *P. kudriavzevii* toward human‐compatible profiles.

## ETHICS STATEMENT

This study did not involve any human participants or animal subjects.

## Supporting information

supporting information‐clean.

## Data Availability

All the data are available in the main text and the Supporting Information.
