# Supplementary material for: Establishment of a genome editing system and expression of heterologous proteins in the nonconventional yeast Pichia kudriavzevii
Source: mLife. 2026 Sep 29;5(5):665–9. doi: 10.1002/mlf2.70104 (PMC13624304; doi:10.1002/mlf2.70104)
Supplement: Supplementary file 1 [file MLF2-5-665-s001.docx]

**Establishment of genome editing system and expression heterologous protein in the non-conventional yeast *Pichia kudriavzevii***

Jiayin Ma^1,2^, Yifan Cui^1,2^, Binru Yin^3^, Cheng Jin^1^*

^1^ State Key Laboratory of Microbial Diversity and Innovative Utilization, Institute of Microbiology, Chinese Academy of Sciences, Beijing 100101, China; ^2^ University of Chinese Academy of Sciences, Beijing, China; ^3^ Beijing CAStar Union Sci-Tech Ltd., Beijing, 100080, China.

Corresponding author: jinc@im.ac.cn

**Materials and Methods**

**Strains, media, and chemicals**

*Escherichia coli* DH5α transformants were cultured in LB broth (Lennox) supplemented with ampicillin (100 μg/mL) at 37℃. *Pichia kudriavzevii* LC375240 strains, including wild-type and derivatives, were cultivated in YPD medium (1% yeast extract, 2% peptone, 2% glucose) at 30℃. Transformants were selected on synthetic dextrose minimal medium (SD; 0.67% yeast nitrogen base, 2% glucose). URA3-auxotrophic strains were screened on SD plates containing uracil (100 μg/mL) and 5-fluoroorotic acid (1 mg/mL, 5-FOA). DNA purification kits (Axygen Scientific), oligonucleotides (Qingke Biotechnology), and chemicals (Sigma-Aldrich; Fisher Scientific) were obtained commercially.

**Plasmid construction**

CRISPR/Cas9 plasmids were assembled by ligating PCR-amplified fragments (KOD FX polymerase, TOYOBO #KFX-101) into linearized backbones using ClonExpress Ultra One Step Cloning Kit (Vazyme Biotech #C115). Three functional cassettes, including Cas9, sgRNA and *Saccharomyces cerevisiae*-derived ARS, were integrated into the backbone. Constructs were amplified in *E. coli* DH5α, and positive clones were confirmed by Sanger sequencing. Validated plasmids were electroporated into *P. kudriavzevii* LC375240 for target gene expression. Primers used in this study are listed in Table S2.

**Yeast transformation**

Mid-log-phase cultures (OD₆₀₀ = 1.3~1.5) were harvested (5,000 × g, 5 min, 4℃), washed twice with ice-cold deionized water, and resuspended in pretreatment buffer (100 mM LiAc, 10 mM DTT, 0.6 M sorbitol, 10 mM Tris-HCl, pH 7.5). Cells were incubated at 30℃, 100 rpm for 30 min, pelleted, washed twice with 1 M ice-cold sorbitol, and resuspended in 1 M sorbitol to generate competent cells. Transformation mixtures contained 80 µL competent cells, 500 ng plasmid DNA, and 1 µg donor DNA, transferred to 2-mm electroporation cuvettes, incubated on ice for 5 min, and pulsed at 2.5 kV, 25 µF, 200 Ω (Gene Pulser Xcell, Bio-Rad). Cells were rescued in 1 mL YPD and 1 M sorbitol at 30℃ for 2 h and plated on selective media.

**Codon optimization**

Cas9 codon usage was optimized based on *P. kudriavzevii* RSCU value. All ORFs of *P. kudriavzevii* were analyzed to identify optimal codons. Rare codons in *Komagataella phaffii*-derived Cas9 were replaced with *P. kudriavzevii*-preferred codons. Optimized sequences were manually refined and synthesized (Shanghai Sangon Biotech).

**mCherry fluorescence measurement**

Recombinant strains were incubated overnight at 30℃, 200 rpm, diluted to OD₆₀₀=0.1 in fresh YPD medium, and grown for 72 h under the same conditions. The supernatant measured fluorescence (Ex 580 nm, Em 610 nm) using a microplate reader (Synergy H4, BioTek). Three biological replicates were performed.

**Human IgG1 Fc fragment purification**

The human IgG1 Fc fragment was captured by affinity chromatography from the supernatant medium of *P. kudriavzevii* fermentations using a HyPur T Protein A 4FF PrePacked Gravity Column (BBI, China). The resin was equilibrated with 150 mM NaCl, 20 mM Na_2_HPO_4_, pH 7 and the supernatant medium was dialyzed using the same buffer. The column was washed with 5 column volumes of the same buffer and the human IgG1 Fc fragment was eluted with 100 mM Glycine-HCl pH 3. The eluted protein was neutralized immediately with 1 M Tris-HCl, pH 8.5.

**SDS-PAGE analysis**

An equivalent of 3 mL of the supernatant obtained by centrifugation of each yeast cell culture was concentrated to 10 μL and mixed with 2.5 μL of 5 × sample buffer containing a reducing reagent (Solarbio, China). This was followed by incubation at 100 °C for more than 10min. Next, 10 μL of the resultant lysate was separated on a 12.5 % polyacrylamide gel using SDS-PAGE (EpiZyme, China) and stained with Supermicroscale Protein Stain (Mei5bio, China).

**Western blotting**

An equivalent of 10 μL purified human IgG1 Fc fragment were analyzed via SDS-PAGE using a 12.5 % polyacrylamide gel (Cat. PG213, EpiZyme, China). The protein were transferred onto PVDF membranes (Cytiva) using a wet transfer system (300 mA, 90 min, 4 °C). Membranes were blocked with 5% non-fat milk in TBST (20 mM Tris–HCl, pH 7.6, 150 mM NaCl, 0.1% Tween-20) for 1 h at room temperature, and incubated overnight at 4 °C with FC of human IgG monoclonal antibody (Proteintech, , USA) diluted in blocking buffer. After three washes with TBST (10 min each), membranes were incubated for 1 h at room temperature with HRP-conjugated secondary antibodies (1:5,000 dilution). Immunoreactive bands were visualized using enhanced chemiluminescence (ECL, Millipore) and imaged with a TANON 5200Multi system (Tanon).

**Chemical analysis of cell wall components**

200 mg of the yeast cells were harvested (5,000 × g, 5 min, 4℃), washed twice with distilled water and were ground in 50 mM NH4HCO3 (pH 8.0) using Disruptor Genie (Scientific Industries) with 0.2 g of glass beads (0.5 mm diameter) for 1 h. The cell wall pellets were collected and treated with 1 M KOH at 70°C for 0.5 h to release alkali-soluble glycoproteins and α-glucans. When the pH of alkali-soluble materials was adjusted to 5.0, α-glucans were precipitated and collected by centrifugation. Glycoproteins in the supernatant were precipitated with 4 volumes of 100% ethanol and dissolved in distilled water. Protein was determined by Lowry protein assay ^1^. Alkali-insoluble components contain β-glucan and chitin. β-glucan, α-glucan and chitin were washed with water for 3 times, then 200 μL of 6 M HCl was added and hydrolyzed at 100 °C for 2 hours (the acid hydrolysis of β-glucan and α-glucan yields glucose, and the acid hydrolysis of chitin yields GlcN). The solution was vacuum-dried, and HCl was removed. 200 μL of water was added to dissolve the monosaccharides, and the amount of glucose was determined by the phenol-sulfuric acid method ^2^ to determine the content of β-glucan and α-glucan. The amount of GlcN was determined by Lee's method ^3^ to determine the content of chitin.

**q-PCR**

Total RNA was extracted from mid-log-phase cultures using Yeast Total RNA Rapid Extraction Kit (ZOMANBIO, China). cDNA was synthesized from 1 μg RNA with FastKing cDNA First Strand Synthesis Kit (TIANGEN, China). qPCR was performed using SYBR FAST qPCR Master Mix (KAPA Biosystems, USA) on a Bio-Rad CFX96 system in 20 μL reactions (200 nM primers). Cycling: 95℃ 3 min; 40 cycles of 95℃ 3 s, 60℃ 30 s; followed by melting curve analysis. *ACT1* served as an internal reference. Experiments were performed in biological triplicates with technical replicates.

**Table S1.** Genetic elements/DNA sequences used in this study

| **Genetic elements/DNA** | **Sequences** |
| --- | --- |
| ScARS | gatcgccaacaaatactaccttttatcttgctcttcctgctctcaggtattaatgccgaattgtttcatcttgtctgtgtagaagaccacacacgaaaatcctgtgattttacattttacttatcgttaatcgaatgtatatctatttaatctgcttttcttgtctaataaatatatatgtaaagtacgctttttgttgaaattttttaaacctttgtttatttttttttcttcattccgtaactcttctaccttctttatttactttctaaaatccaaatacaaaacataaaaataaataaacacagagtaaattcccaaattattccatcattaaaagatacgaggcgcgtgtaagttacaggcaagcgatc |
| KlARS | GCAACATCTTTGGATAATATCAGAATGAGAAAGAACAGATACGCAGTACGTTTTTTGGTGAGCTCTTTGCACTTCTTTAGTTCTTTCCATCAATATCAGTTGCTTATGCACTTATGACTAATATTGATGTTTAACTTCAATATCTTTAAACTTTTGTTCTTCCCGACGTTCATTAAGAATACTAATACACTTTAATAATTAGTTTAATATTTGTTTCTATATAATGACATTTAAttaaaaaagataaaataTAAAAACATCATAATAACTCACCAGAGGTtaagaacaaaaaaacaaattaGATATCTGCTAATCCAATATAGTTAAATCAATCTTTCCTTGGTATAATGGGTATATTACATATATTTCAAGGACCGACACTCCTACCAAATATCTAAAATTTACCATATTAACATAACATGTATATAAACGTCAAATCATAATCAGCACTA |
| PpARS | TCGAGATAAGCTGGGGGAACATTCGCGAAAATGAAACAAGTCGGCTGTTATAGTATATTTATTATAATATTGAAAGATCTCAAAAGACTACTTATTTTTGAATGAACCAAGTATGAAATCAACCTATTTGGGGTTGACCAAAATAAGTAAATATTAATTGTCGA |
| KmARS | GATCCAAGTCTGAAGGTTGGTTTGGCACTAACTTTACTCTTGTTATATTCAGAATTGTATCAAGTTTATTTGTTAGAGTGGAGCCTTTTTTTATCCGTAACACTTTTTCCCTGCTCCATTTTGAAAAACGATTTCAGGCCATCTTGGCTATTCCGAATGAATTTGGAATATGTTTaaattaataaaaataaaataaaataaaattaaaattaaattgaattaaattaaattaaattaaattaaattaaataaaaataaatacaaCCAATACAACATGGTAATATTCTTGCATCGTAATGAATATTAAATATCACTTTATTAATCTCATTAtgttttattgtttttgtaaGGACTTTAATATATTTGAATCAATATTCTTTCATTTACTAGTACTTTTTTTATATGACTAAAATTGTTACACATTGGACTGACAGttatttttaaaatttatgatttattcttattttatATCTTTAAAAGTAGAAATATTATACGGACGCTTTGAATGCAATTGACAACTTATCTTACTAGTGTGAATCAACCCTATCGATGTAGTACTCTCAAAATACGGCCTTCTTGATAAAGTGTTAAATTCATTTGGGTAATGATTTTTCGAAAACCACATTGAATGAACGatttaaataaatatagGATGCAAGAGCATTTTAATAAttcagaaacaaacaaattATTAAACAGGAGCAGTTGAACGGTATGTTAACGAGTTTTGTAAAGGGTGAGTACATTTATAGCTCTATTGGACATAATAAATACATATAAATAGTATTTTTTGACCCTCTATGAAGATGGCTTACCAGCAACTTATGTCTTTTAATTCACGTGACTACtaaacaataaaatatgttatttaaaaaatatttatttaaaTTTTTAAACTATTATAGATTATTTGTGAATgcattattttttaatttattaaTTAAAAGAATTGCTATTTACTtaaaataagaataaaagctttttatttttttaaaagaaaaatatattaaaaaCACTTTTCCGAAAGTTAAAAtaattttatatttatCGGTAGCTGCAATTTATAGACataatattttatattttttaaaatttattattattttgtttgaaataATAACGTCTGTGAGTGTTTAAGGTGAACTAAGACTGAAAAAGTACATAATTTTTGTTAATTTTATGATATGATC |
| *HsCas9* | ATGGACAAGAAGTACTCCATTGGGCTCGATATCGGCACAAACAGCGTCGGCTGGGCCGTCATTACGGACGAGTACAAGGTGCCGAGCAAAAAATTCAAAGTTCTGGGCAATACCGATCGCCACAGCATAAAGAAGAACCTCATTGGCGCCCTCCTGTTCGACTCCGGGGAGACGGCCGAAGCCACGCGGCTCAAAAGAACAGCACGGCGCAGATATACCCGCAGAAAGAATCGGATCTGCTACCTGCAGGAGATCTTTAGTAATGAGATGGCTAAGGTGGATGACTCTTTCTTCCATAGGCTGGAGGAGTCCTTTTTGGTGGAGGAGGATAAAAAGCACGAGCGCCACCCAATCTTTGGCAATATCGTGGACGAGGTGGCGTACCATGAAAAGTACCCAACCATATATCATCTGAGGAAGAAGCTTGTAGACAGTACTGATAAGGCTGACTTGCGGTTGATCTATCTCGCGCTGGCGCATATGATCAAATTTCGGGGACACTTCCTCATCGAGGGGGACCTGAACCCAGACAACAGCGATGTCGACAAACTCTTTATCCAACTGGTTCAGACTTACAATCAGCTTTTCGAAGAGAACCCGATCAACGCATCCGGAGTTGACGCCAAAGCAATCCTGAGCGCTAGGCTGTCCAAATCCCGGCGGCTCGAAAACCTCATCGCACAGCTCCCTGGGGAGAAGAAGAACGGCCTGTTTGGTAATCTTATCGCCCTGTCACTCGGGCTGACCCCCAACTTTAAATCTAACTTCGACCTGGCCGAAGATGCCAAGCTTCAACTGAGCAAAGACACCTACGATGATGATCTCGACAATCTGCTGGCCCAGATCGGCGACCAGTACGCAGACCTTTTTTTGGCGGCAAAGAACCTGTCAGACGCCATTCTGCTGAGTGATATTCTGCGAGTGAACACGGAGATCACCAAAGCTCCGCTGAGCGCTAGTATGATCAAGCGCTATGATGAGCACCACCAAGACTTGACTTTGCTGAAGGCCCTTGTCAGACAGCAACTGCCTGAGAAGTACAAGGAAATTTTCTTCGATCAGTCTAAAAATGGCTACGCCGGATACATTGACGGCGGAGCAAGCCAGGAGGAATTTTACAAATTTATTAAGCCCATCTTGGAAAAAATGGACGGCACCGAGGAGCTGCTGGTAAAGCTTAACAGAGAAGATCTGTTGCGCAAACAGCGCACTTTCGACAATGGAAGCATCCCCCACCAGATTCACCTGGGCGAACTGCACGCTATCCTCAGGCGGCAAGAGGATTTCTACCCCTTTTTGAAAGATAACAGGGAAAAGATTGAGAAAATCCTCACATTTCGGATACCCTACTATGTAGGCCCCCTCGCCCGGGGAAATTCCAGATTCGCGTGGATGACTCGCAAATCAGAAGAGACCATCACTCCCTGGAACTTCGAGGAAGTCGTGGATAAGGGGGCCTCTGCCCAGTCCTTCATCGAAAGGATGACTAACTTTGATAAAAATCTGCCTAACGAAAAGGTGCTTCCTAAACACTCTCTGCTGTACGAGTACTTCACAGTTTATAACGAGCTCACCAAGGTCAAATACGTCACAGAAGGGATGAGAAAGCCAGCATTCCTGTCTGGAGAGCAGAAGAAAGCTATCGTGGACCTCCTCTTCAAGACGAACCGGAAAGTTACCGTGAAACAGCTCAAAGAAGACTATTTCAAAAAGATTGAATGTTTCGACTCTGTTGAAATCAGCGGAGTGGAGGATCGCTTCAACGCATCCCTGGGAACGTATCACGATCTCCTGAAAATCATTAAAGACAAGGACTTCCTGGACAATGAGGAGAACGAGGACATTCTTGAGGACATTGTCCTCACCCTTACGTTGTTTGAAGATAGGGAGATGATTGAAGAACGCTTGAAAACTTACGCTCATCTCTTCGACGACAAAGTCATGAAACAGCTCAAGAGGCGCCGATATACAGGATGGGGGCGGCTGTCAAGAAAACTGATCAATGGGATCCGAGACAAGCAGAGTGGAAAGACAATCCTGGATTTTCTTAAGTCCGATGGATTTGCCAACCGGAACTTCATGCAGTTGATCCATGATGACTCTCTCACCTTTAAGGAGGACATCCAGAAAGCACAAGTTTCTGGCCAGGGGGACAGTCTTCACGAGCACATCGCTAATCTTGCAGGTAGCCCAGCTATCAAAAAGGGAATACTGCAGACCGTTAAGGTCGTGGATGAACTCGTCAAAGTAATGGGAAGGCATAAGCCCGAGAATATCGTTATCGAGATGGCCCGAGAGAACCAAACTACCCAGAAGGGACAGAAGAACAGTAGGGAAAGGATGAAGAGGATTGAAGAGGGTATAAAAGAACTGGGGTCCCAAATCCTTAAGGAACACCCAGTTGAAAACACCCAGCTTCAGAATGAGAAGCTCTACCTGTACTACCTGCAGAACGGCAGGGACATGTACGTGGATCAGGAACTGGACATCAATCGGCTCTCCGACTACGACGTGGATCATATCGTGCCCCAGTCTTTTCTCAAAGATGATTCTATTGATAATAAAGTGTTGACAAGATCCGATAAAAATAGAGGGAAGAGTGATAACGTCCCCTCAGAAGAAGTTGTCAAGAAAATGAAAAATTATTGGCGGCAGCTGCTGAACGCCAAACTGATCACACAACGGAAGTTCGATAATCTGACTAAGGCTGAACGAGGTGGCCTGTCTGAGTTGGATAAAGCCGGCTTCATCAAAAGGCAGCTTGTTGAGACACGCCAGATCACCAAGCACGTGGCCCAAATTCTCGATTCACGCATGAACACCAAGTACGATGAAAATGACAAACTGATTCGAGAGGTGAAAGTTATTACTCTGAAGTCTAAGCTGGTCTCAGATTTCAGAAAGGACTTTCAGTTTTATAAGGTGAGAGAGATCAACAATTACCACCATGCGCATGATGCCTACCTGAATGCAGTGGTAGGCACTGCACTTATCAAAAAATATCCCAAGCTTGAATCTGAATTTGTTTACGGAGACTATAAAGTGTACGATGTTAGGAAAATGATCGCAAAGTCTGAGCAGGAAATAGGCAAGGCCACCGCTAAGTACTTCTTTTACAGCAATATTATGAATTTTTTCAAGACCGAGATTACACTGGCCAATGGAGAGATTCGGAAGCGACCACTTATCGAAACAAACGGAGAAACAGGAGAAATCGTGTGGGACAAGGGTAGGGATTTCGCGACAGTCCGGAAGGTCCTGTCCATGCCGCAGGTGAACATCGTTAAAAAGACCGAAGTACAGACCGGAGGCTTCTCCAAGGAAAGTATCCTCCCGAAAAGGAACAGCGACAAGCTGATCGCACGCAAAAAAGATTGGGACCCCAAGAAATACGGCGGATTCGATTCTCCTACAGTCGCTTACAGTGTACTGGTTGTGGCCAAAGTGGAGAAAGGGAAGTCTAAAAAACTCAAAAGCGTCAAGGAACTGCTGGGCATCACAATCATGGAGCGATCAAGCTTCGAAAAAAACCCCATCGACTTTCTCGAGGCGAAAGGATATAAAGAGGTCAAAAAAGACCTCATCATTAAGCTTCCCAAGTACTCTCTCTTTGAGCTTGAAAACGGCCGGAAACGAATGCTCGCTAGTGCGGGCGAGCTGCAGAAAGGTAACGAGCTGGCACTGCCCTCTAAATACGTTAATTTCTTGTATCTGGCCAGCCACTATGAAAAGCTCAAAGGGTCTCCCGAAGATAATGAGCAGAAGCAGCTGTTCGTGGAACAACACAAACACTACCTTGATGAGATCATCGAGCAAATAAGCGAATTCTCCAAAAGAGTGATCCTCGCCGACGCTAACCTCGATAAGGTGCTTTCTGCTTACAATAAGCACAGGGATAAGCCCATCAGGGAGCAGGCAGAAAACATTATCCACTTGTTTACTCTGACCAACTTGGGCGCGCCTGCAGCCTTCAAGTACTTCGACACCACCATAGACAGAAAGCGGTACACCTCTACAAAGGAGGTCCTGGACGCCACACTGATTCATCAGTCAATTACGGGGCTCTATGAAACAAGAATCGACCTCTCTCAGCTCGGTGGAGACTAA |
| *PKCas9* | ATGGATAAAAAATATTCTATTGGTTTGGATATTGGTACAAATTCTGTTGGTTGGGCAGTTATTACAGACGAATATAAAGTTCCTTCTAAAAAATTTAAAGTTTTGGGTAATACAGATAGACATTCTATCAAAAAAAATTTGATTGGTGCATTGTTGTTTGATTCTGGTGAAACAGCAGAAGCAACAAGATTAAAGAGAACAGCTAGAAGGAGATATACTAGAAGAAAAAATAGAATCTGCTACTTGCAAGAAATTTTCTCTAACGAGATGGCAAAGGTTGACGATTCTTTCTTTCACAGATTAGAAGAATCTTTTTTGGTTGAAGAAGACAAAAAACATGAAAGACATCCTATTTTTGGTAATATTGTTGATGAGGTTGCATACCATGAGAAATATCCAACTATTTACCATTTAAGGAAGAAATTGGTTGATTCTACAGATAAAGCAGATTTGAGATTGATTTATTTGGCATTGGCACATATGATTAAATTTAGAGGTCATTTTTTGATTGAGGGAGATTTGAACCCTGATAACTCTGACGTCGATAAGTTATTCATTCAATTGGTTCAGACATACAATCAATTGTTTGAAGAAAATCCAATTAATGCATCTGGTGTTGATGCAAAAGCAATTTTGTCTGCAAGATTGTCTAAATCTAGAAGATTGGAAAATTTGATTGCACAATTGCCTGGTGAAAAAAAAAATGGTTTGTTTGGTAATTTGATCGCATTGTCTTTGGGTTTGACACCAAATTTTAAATCTAATTTTGATTTGGCAGAAGATGCAAAATTGCAGTTGTCTAAAGACACATATGATGACGATTTGGATAATTTGTTGGCACAAATTGGTGATCAATATGCAGATTTGTTTTTGGCAGCAAAAAATTTGTCTGACGCAATCTTGTTGTCTGATATTTTGAGAGTTAATACAGAAATTACAAAGGCTCCATTGTCTGCATCTATGATTAAAAGATATGATGAACATCATCAAGATTTGACATTGTTGAAGGCATTGGTCAGACAGCAATTGCCTGAAAAATACAAAGAAATCTTCTTCGACCAATCTAAAAATGGTTATGCTGGTTATATCGACGGTGGTGCATCTCAAGAAGAGTTTTATAAATTTATTAAGCCAATTTTGGAAAAAATGGATGGAACAGAAGAATTGTTAGTCAAATTGAATAGAGAAGATTTGTTGAGAAAACAGAGAACTTTCGATAATGGTTCTATTCCACATCAAATTCATTTGGGTGAGTTGCATGCAATTTTGAGAAGGCAAGAGGATTTTTACCCATTCTTGAAGGATAACAGAGAGAAAATTGAGAAGATTTTAACTTTTAGGATTCCATATTATGTTGGTCCATTGGCAAGAGGTAATTCTAGATTTGCATGGATGACAAGAAAATCTGAAGAAACAATTACACCATGGAATTTTGAAGAAGTTGTCGACAAGGGTGCATCAGCACAATCATTTATTGAGAGGATGACTAATTTCGATAAAAATTTGCCAAATGAAAAAGTTTTGCCAAAGCATTCTTTGTTGTATGAATATTTTACAGTTTACAATGAATTAACAAAGGTTAAATATGTTACAGAAGGTATGAGAAAACCTGCATTTTTGTCTGGTGAACAAAAAAAAGCAATTGTTGATTTGTTGTTCAAAACTAATAGAAAAGTTACTGTTAAGCAATTGAAAGAAGACTATTTCAAGAAAATTGAATGTTTTGATTCTGTTGAAATTTCTGGTGTCGAAGATAGATTCAATGCATCTTTGGGTACATACCACGATTTATTAAAGATTATCAAGGATAAAGATTTCTTGGACAACGAAGAGAATGAGGATATTTTGGAAGATATTGTTTTAACATTGACATTGTTCGAGGATAGAGAAATGATTGAAGAAAGATTGAAAACATACGCACATTTGTTTGATGACAAGGTTATGAAACAATTAAAAAGGAGAAGATATACTGGTTGGGGAAGGTTGTCTAGGAAATTAATCAATGGTATTAGGGATAAACAATCTGGTAAAACAATCTTGGATTTCTTGAAATCTGATGGATTTGCAAATAGAAATTTCATGCAATTGATTCATGACGACTCTTTGACATTTAAAGAAGATATTCAAAAAGCACAAGTTTCTGGTCAAGGTGATTCTTTGCATGAACATATTGCAAATTTGGCTGGTTCTCCTGCAATTAAAAAAGGTATTTTGCAAACAGTTAAAGTTGTTGATGAATTGGTTAAAGTTATGGGTAGACATAAACCTGAAAATATTGTTATTGAAATGGCAAGAGAAAATCAGACTACACAAAAAGGTCAGAAAAACTCAAGAGAAAGAATGAAAAGAATTGAAGAGGGTATTAAAGAATTGGGTTCACAGATTTTGAAAGAACATCCTGTCGAAAATACACAATTACAAAATGAAAAATTATATTTGTATTATTTGCAAAATGGTAGAGATATGTATGTTGATCAAGAGTTAGACATTAATAGATTGTCTGACTATGATGTTGATCACATCGTTCCACAATCATTTTTGAAAGATGATTCAATTGACAATAAAGTTTTGACAAGGTCTGATAAAAATAGGGGAAAGTCTGATAATGTTCCATCAGAAGAGGTTGTTAAAAAGATGAAAAACTACTGGAGACAATTGTTAAATGCAAAGTTGATCACACAAAGAAAGTTTGATAATTTGACAAAAGCAGAAAGAGGTGGTTTGTCTGAATTGGATAAAGCTGGTTTTATTAAAAGACAATTAGTTGAGACAAGGCAAATTACAAAACATGTTGCACAGATTTTGGATTCTAGGATGAATACAAAGTATGACGAAAATGATAAATTGATCAGAGAAGTTAAAGTTATCACATTGAAATCAAAATTGGTCTCAGACTTCAGAAAAGACTTTCAGTTTTACAAAGTTAGGGAGATTAATAATTATCATCATGCACATGATGCATATTTGAATGCAGTTGTTGGTACAGCATTGATTAAAAAATATCCAAAGTTGGAATCTGAGTTTGTTTATGGTGACTATAAAGTCTATGATGTCAGAAAAATGATTGCAAAGTCTGAACAAGAAATTGGTAAAGCAACTGCTAAGTATTTCTTCTATTCTAACATCATGAATTTTTTTAAAACTGAAATTACTTTGGCAAATGGTGAAATTAGGAAAAGACCATTGATTGAAACAAACGGTGAAACTGGTGAAATTGTTTGGGATAAAGGTAGAGATTTTGCAACAGTTAGAAAAGTTTTATCTATGCCACAAGTTAATATTGTCAAAAAGACAGAAGTTCAAACTGGTGGTTTTTCTAAAGAATCTATCTTGCCAAAAAGAAATTCTGATAAGTTGATTGCAAGAAAAAAAGATTGGGATCCAAAGAAATACGGTGGTTTTGATTCTCCAACAGTTGCTTACTCTGTTTTGGTCGTTGCAAAAGTCGAAAAGGGTAAGTCTAAAAAGTTAAAATCAGTTAAGGAATTATTAGGTATTACTATTATGGAAAGGTCTTCTTTTGAGAAAAATCCAATTGATTTTTTGGAGGCTAAGGGATACAAAGAAGTCAAGAAAGATTTGATCATCAAATTACCTAAATACTCTTTATTCGAATTGGAAAATGGTAGAAAAAGAATGTTGGCATCTGCTGGTGAATTGCAAAAAGGTAATGAATTGGCATTGCCATCTAAATACGTTAATTTTTTGTATTTGGCATCTCATTATGAAAAATTGAAAGGTTCTCCTGAAGATAACGAGCAGAAGCAATTGTTTGTTGAACAGCATAAACATTATTTAGACGAAATTATTGAACAAATTTCTGAGTTTTCTAAGAGAGTTATTTTGGCAGATGCAAATTTGGACAAAGTTTTGTCTGCTTATAATAAACATAGAGATAAACCAATCAGAGAGCAAGCAGAAAATATTATTCATTTGTTTACATTGACAAATTTGGGTGCACCTGCAGCATTCAAATACTTTGACACAACAATCGATAGGAAAAGGTATACATCAACAAAAGAAGTTTTGGATGCAACATTGATTCATCAATCTATTACTGGTTTGTATGAAACAAGAATTGATTTGTCTCAATTGGGTGGTGATTAA |
| *mCherry* | ATGGTTTCTAAGGGTGAAGAAGATAACATGGCTATCATCAAGGAATTCATGAGATTCAAGGTTCACATGGAAGGTTCTGTTAACGGTCACGAATTCGAAATCGAAGGTGAAGGTGAAGGTAGACCATACGAAGGTACTCAAACTGCTAAGTTGAAGGTTACTAAGGGTGGTCCATTGCCATTCGCTTGGGATATCTTGTCTCCACAATTCATGTACGGTTCTAAGGCTTACGTTAAGCACCCAGCTGATATCCCAGATTACTTGAAGTTGTCTTTCCCAGAAGGTTTCAAGTGGGAAAGAGTTATGAACTTCGAAGATGGTGGTGTTGTTACTGTTACTCAAGATTCTTCTTTGCAAGATGGTGAATTCATCTACAAGGTTAAGTTGAGAGGTACTAACTTCCCATCTGATGGTCCAGTTATGCAAAAGAAGACTATGGGTTGGGAAGCTTCTTCTGAAAGAATGTACCCAGAAGATGGTGCTTTGAAGGGTGAAATCAAGCAAAGATTGAAGTTGAAGGATGGTGGTCACTACGATGCTGAAGTTAAGACTACTTACAAGGCTAAGAAGCCAGTTCAATTGCCAGGTGCTTACAACGTTAACATCAAGTTGGATATCACTTCTCACAACGAAGATTACACTATCGTTGAACAATACGAAAGAGCTGAAGGTAGACACTCTACTGGTGGTATGGATGAATTGTACAAGcatcatcatcatcatcatTAA |
| human IgG1 Fc fragment | ATGGAACCAAAATCATGTGATAAAACACATACATGTCCACCATGTCCAGCACCAGAATTGTTGGGTGGTCCATCAGTTTTTTTGTTTCCACCAAAACCAAAAGATACATTGATGATTTCAAGAACACCAGAAGTTACATGTGTTGTTGTTGATGTTTCACATGAAGATCCAGAAGTTAAATTTAATTGGTATGTTGATGGTGTTGAAGTTCATAATGCAAAAACAAAACCAAGAGAAGAACAATATAATTCAACATATAGAGTTGTTTCAGTTTTGACAGTTTTGCATCAAGATTGGTTGAATGGTAAAGAATATAAATGTAAAGTTTCAAATAAAGCATTGCCAGCACCAATTGAAAAAACAATTTCAAAAGCAAAAGGTCAACCAAGAGAACCACAAGTTTATACATTGCCACCATCAAGAGATGAATTGACAAAAAATCAAGTTTCATTGACATGTTTGGTTAAAGGTTTTTATCCATCAGATATTGCAGTTGAATGGGAATCAAATGGTCAACCAGAAAATAATTATAAAACAACACCACCAGTTTTGGATTCAGATGGTTCATTTTTTTTGTATTCAAAATTGACAGTTGATAAATCAAGATGGCAACAAGGTAATGTTTTTTCATGTTCAGTTATGCATGAAGCATTGCATAATCATTATACACAAAAATCATTGTCATTGTCACCAGGTAAAcatcatcatcatcatcatTAA |
| signal peptide of the *SED1* | CAATTCAAGTACTTAGCACCATTAGCTTTAGCAGGTTCTGCTGTCGCAGCTTTC |
| **Promoters (Gene_ID)** | **sequences** |
| P_HTX1_ | TGTTGTAGTTTTAATATAGTTTGAGTATGAGATGGAACTCAGAACGAAGGAATTATCACCAGTTTATATATTCTGAGGAAAGGGTGTGTCCTAAATTGGACAGTCACGATGGCAATAAACGCTCAGCCAATCAGAATGCAGGAGCCATAAATTGTTGTATTATTGCTGCAAGATTTATGTGGGTTCACATTCCACTGAATGGTTTTCACTGTAGAATTGGTGTCCTAGTTGTTATGTTTCGAGATGTTTTCAAGAAAAACTAAAATGCACAAACTGACCAATAATGTGCCGTCGCGCTTGGTACAAACGTCAGGATTGCCACCACTTTTTTCGCACTCTGGTACAAAAGTTCGCACTTCCCACTCGTATGTAACGAAAAACAGAGCAGTCTATCCAGAACGAGACAAATTAGCGCGTACTGTCCCATTCCATAAGGTATCATAGGAAACGAGAGTCCTCCCCCCATCACGTATATATAAACACACTGATATCCCACATCCGCTTGTCACCAAACTAATACATCCAGTTCAAGTTACCTAAACAAATCAAA |
| P_TDH3_ | tatggatatggagatgaatttgaatttagatttgggtcttgatttggggttggaattaaaaggggataacaatgagggttttcctgttgatttaaacaatggacgtgggaggtgattgatttaacctgatccaaaaggggtatgtctattttttagagagtgtttttgtgtcaaattatggtagaatgtgtaaagtagtataaactttcctctcaaatgacgaggtttaaaacaccccccgggtgagccgagccgagaatggggcaattgttcaatgtgaaatagaagtatcgagtgagaaacttgggtgttggccagccaagggggggggggggaaggaaaatggcgcgaatgctcaggtgagattgttttggaattgggtgaagcgaggaaatgagcgacccggaggttgtgactttagtggcggaggaggacggaggaaaagccaagagggaagtgtatataaggggagcaatttgccaccaggatagaattggatgagttataattctactgtatttattgtataatttatttctccttttgtatcaaacacattacaaaacacacaaaacacacaaacaaacacaattacaaaaa |
| P_TEF1_ | tttgaaacatcatgaaaactgtttcaccctctgtgaagcataaacactagaaagccaatgaagagctctacaagcctcatatgggttcaatgggtctgcaatgaccgcatacgggcttggacaattaccttctattgaatttctgagaagagatacatctgaccagcaatgtaagcagacaatcccaattctgtaaacaacctctttgtccataattccccatcagaagagtgaaaaatgccctcaaaatgcatgcgccacacccacctctcaactgcactgcgccacatctgagggtcctttcaggggtcgactaccccggacacctcgcagaggagcgacgtcacgtacttttaaaatggcagagacgcgcagtttcttgaagaaaggataaaaatgaaatggtgcggaaatgcgaaaatgatgaaaaattttcttggtggcgaggaaattgagtgcaataattggcacgaggttgttgccacccgagtgtgagtatatatcctagtttctgcacttttcttcttcttttctttacgttttcttttcaacttttttttactttttccttcaacagacaaatctaacttatatatcaca |
| P_RNR2_ | aagaatacaacaaaagaatttgtgtttctacagatgttttcggtagaggtattgatatcgaaagaatcaacttggccatcaactacgatatgccaagtgaagcagatcagtacttgcacagagttggtagagcaggtagattcggtactaaaggtttggctgtttcctttatctctaatgaaaaggatgaagaaatcttgaacaagatccaagataggtttgatgttaagattcaagagttccctgaagaaggtgttgatccttctacttacatgaacacttaattgacattgatctgtcatagttttgtggttgttttttttttgaagttatatacatttattaatatatctttatatatatattgtccaaatccatcggaaagggtctactgtaatacaatcccatatagtagaatgtactcttatatgctgcaatgaggacttgctctggagatatcgttgataagggactaccttggcttccaacgcgttggtcaacaaactgaccttgaagtgcaaatcccgacaggcacgcgtgagatatcggaggtgaatgcacctccaaatcagacaacgcgagcaaacgacgcgtaacacaaggcttgacgcgtacgatgcagacttttcatagtgtgtcgccactgagcgggaggcgggttataaaagaaacgtgtttggcgtatcttttctcttctctggatttgttcttctacttcctttactcctcatcaaatcgtctctccctcgtcactcaccacaactagtccgacactatctgatatacccttattgctaact |
| P_RPR1_ | AGTCCAAAAGGCTCCACCAAAAAAAAGGTAAAAAACACGGCCACGTGACCCACATGTCCACCCGTGCACCGCAACAGTGTTCCCAGCGCTTCCCCCTCCTTTGATACGAGTTTGTGCTGCCGTAGCCTAGCGGTGAAGGGCAGTTCCAAGCGCCCTTTCGAAGCCCGCCGTTGCCAATAGTCCATGCGTACTCGTATACAAAAGGATTCCATTAAGTAATCAATTATTTAAGCGAGTGGGAAATTCGGTGGTATGCGCCGTCCCCGGGCTTTCTCTCTAATAGAGTAGCTCGGTCCCCTGGATAGCTTATTGCTGTCTTCCTCTTGAGAGGTTCCTGGGCGTGCAAGCGCCGGGCAGTTCCACAAGAAA |

**Table S2.** Primers used in this study

| **Primers** | **Sequences (5’ to 3’)** |
| --- | --- |
| RNR2p-F | CCGAGTCGGTGCTTTTTTTTAAGAATACAACAAAAGAATTTG |
| RNR2p-R | AGTTAGCAATAAGGGTATATC |
| TDH3p-F | CCGAGTCGGTGCtatggatatggagatgaatttga |
| TDH3p-R | TTTTTGTAATTGTGTTTGTTTGTG |
| TEF1p-F | CCGAGTCGGTGCtttgaaacatcatgaaaactgttt |
| TEF1p-R | TGTGATATATAAGTTAGATTTGTC |
| HTX1p-F | TGTTGTAGTTTTAATATAGTTTGAG |
| HTX1p-R | TTTGATTTGTTTAGGTAACTTGAA |
| CYC1t-F | GTGAGCTGATAGTTTCTTC |
| CYC1t-R | GGTAGTATTTGTTGGCGATCTTTACGTTTTCTTGTCGTGTG |
| ura3-gRNA-cassette-F | AGTCCAAAAGGCTCCACC |
| ura3-gRNA-cassette-R | AATTCTTTTGTTGTATTCTTAAAAAAAAGCACCGACTCGGTG |
| ScARS-F | ACACGACAAGAAAACGTAAAGATCGCCAACAAATACTACC |
| ScARS-R | GATCGCTTGCCTGTAACTTAC |
| KlARS-F | ACACGACAAGAAAACGTAAAGCAACATCTTTGGATAATATCAG |
| KlARS-R | TAGTGCTGATTATGATTTGACG |
| PpARS-F | ACACGACAAGAAAACGTAAATCGAGATAAGCTGGGGGAAC |
| PpARS-R | TCGACAATTAATATTTACTTATTTTG |
| KmARS-F | ACACGACAAGAAAACGTAAAGATCCAAGTCTGAAGGTTGG |
| KmARS-R | GATCATATCATAAAATTAACAAAAAT |
| PkCas9-F | TAAAACTACAACACGAAACGATGCCAAAAAAGAAAAGAAAAGTTG |
| PkCas9-R | AAACTGTAAAGACTTCCCGTTTAAACTTTCCTTTTCTTTTTTGGATC |
| HsCas9-F | TAAAACTACAACACGAAACGATGGACAAGAAGTACTCCATTGG |
| HsCas9-R | AAACTGTAAAGACTTCCCGTTTAGTCTCCACCGAGCTGAG |
| ura3-upstream-F | GCCACAAATTAGGTTGAGGA |
| ura3-upstream-R | ATCAAATCTTTGTGTAAGAACCTTGACAAACAAACTACTTTA |
| ura3-downstream-F | AAGTAGTTTGTTTGTCAAGGTTCTTACACAAAGATTTGATAC |
| ura3-downstream-R | GCATGGTTTTTAAGCAGAAG |
| ura3-check-F | AATACAAGTGATAAAGTTGAAGG |
| ura3-check-R | TTTTTAAGCAGAAGGGAGAAAC |
| mCherry-F | ATGGTTTCTAAGGGTGAAGAAG |
| mCherry-R | TTAATGATGATGATGATGATGCT |
| hFc-F | ATGGAACCAAAATCATGTGATAA |
| hFc-R | TTAATGATGATGATGATGATGTTT |
| act1-q-F | GGTAGACCAAGACATCAAGG |
| act1-q-R | CCATATCATCCCAGTTGGTG |
| Fc-q-F | GTGGTCCATCAGTTTTTTTGTT |
| Fc-q-R | TCAACACCATCAACATACCAAT |
| mCherry-q-F | GAGATTCAAGGTTCACATGGA |
| mCherry-q-R | ATCCCAAGCGAATGGCAATG |

**Figure S1 Construction of CRISPR/Cas9 genome editing plasmid in *P. kudriavzevii*.** (a) Growth phenotype of transformants carrying plasmids with ARS sequences from different yeasts; (b) Heatmap of codon usage frequency in *P. kudriavzevii*; and (c) Schematic representation of the genome editing plasmid in *P*. *kudriavzevii*.

**Figure S2 Analysis of *ura3-*deletion mutants.** (a) PCR-based validation of *ura3* locus disruption in *P. kudriavzevii*. M: DNA marker; lanes 1-20, transformants; lanes 21-23, wild-type. The wild-type band is 2.5 kb, and the knockout band is 1.7 kb. Heterozygous mutants display both bands. (b) Growth assay on SD medium lacking uracil. The *ura3* mutants failed to grow. (c) Complementation with uracil restored growth of *ura3* mutants.

**Figure S3. Validation of genome editing at the *KU70* and *msds* loci.** (A) PCR analysis of *Ku70* deletion. The wild-type band is 3510-bp, and the knockout band is 1500-bp. M: DNA marker; lanes 1-21, transformants; lanes 22-24, wild-type controls.

**Table S3. Analysis of the components of the cell wall**

**Figure S4** RT-qPCR of mCherry and human IgG1 Fc fragment. Error bars represent mean ± SD of three biological replicates.

**References:**

1. Damveld R A, Arentshorst M, VanKuyk P A, Klis F M, van den Hondel C A M J, Ram A F J. Characterisation of CwpA, a putative glycosylphosphatidylinositol-anchored cell wall mannoprotein in the filamentous fungus *Aspergillus niger*. Fung Genet Biol. 2005; 42(10): 873-885.
2. DUBOIS M, GILLES K, HAMILTON J K, REBERS P A, SMITH F. A colorimetric method for the determination of sugars. Nature. 1951; 168(4265): 167.
3. Lee J I, Yu Y M, Rho Y M, Park B C, Choi J H, Park H et al. Differential expression of the *chsE* gene encoding a chitin synthase of *Aspergillus nidulans* in response to developmental status and growth conditions. FEMS Microbiol Lett. 2005; 249(1): 121-129.
